# To the Editors of the Medical and Physical Journal

**Published:** 1800-05

**Authors:** M. F. Wagstaffe

**Affiliations:** Southwark


### *To the Editors of the Medical and Phyfical Journal.*

GENTLEMEN,

IF you think the following cafe of a fuccefsful termination of an adhefion of the placenta after fifty-three hours, fuitable for infertion in your valuable Journal, it is much at your fervice.

*Southwark,* Your's, refpectfully,

*March* 12, 1800. M. F. WAGSTAFFE.

---

ON the 21ft of December, 1799, I was defired to vifit a lady, who believed herfelf advanced to the feventh month of pregnancy, but was very unwell. She had not been pregnant before during the fpace of eight years. I found her labouring under œdema of the lower extremities to a very confiderable and painful degree, extending almoft to the region of the ftomach, which continued till after delivery.

On the 26th I was fent for, and found her in labour, the os uteri confiderably dilated, and the prefentation natural. In about two hours the pains left her, and did not return (except trifling grinding pains) till the 1ft of January, 1800, about two in the morning. Soon after fhe importuned the nurfe to fend for me, which fhe poftponed till near fix. When I arrived, I found two children had been expelled by natural efforts; the nurfe had divided the funes umbilicales, after fecuring them by ligatures. I immediately made a flight effort on the funes to facilitate the expulfion of the placentæ, but found a fteady uniform extracting force inadequate to the purpofe. Having waited near two hours from the birth of the children, during which I had made frequent unavailing efforts by the funes, I introduced my hand, but found both placentæ adhering firmly to the uterus; my patient being then in an exhaufted ftate, attended with frequent faintings, I thought it more prudent not to ufe violent means, the more particularly as there was no difpofition to hæmorrhage, but defired fhe might be furnifhed with fmall quantities of brandy and water, or red wine, frequently, and gave her a cordial medicine, with a view of ftimulating the uterus into action. I vifited her again in the afternoon, and was informed fhe had frequent faintings during my abfence; but finding there was no difpofition in the uterus either to contraction or hæmorrhage, I left her, defiring the fame plan to be purfued, and information to be fent me if either hæmorrhage or pains fhould come on. I vifited her twice the following day, during which her faintings were not so frequent, the vascular system was excited, but not sufficiently to warrant the necessary operation for the extraction of the placentæ, I therefore ordered the more frequent exhibition of stimuli, and had the pleasure, the following morning, of finding that all unpleasant symptoms had disappeared, and my patient much recruited; I therefore considered that now was the time to assist Nature. Upon introducing the hand, I found one placenta (very small) had separated from the uterus, and was readily extracted; the other, which was extremely large, still adhered to the uterus, but was separated without much difficulty. Fifty-three hours elapsed from the birth of the children to the extraction of the placentæ; and what appeared particularly singular to me was, that no disposition to hæmorrhage appeared during that time. I continued the cordial tonic plan, and had the satisfaction to see my patient able to quit her chamber at the expiration of three weeks, and remain perfectly well.

---

